# Association between Influenza Vaccine Administration and Primary Care Consultations for Respiratory Infections: Sentinel Network Study of Five Seasons (2014/2015–2018/2019) in the UK

**DOI:** 10.3390/ijerph18020523

**Published:** 2021-01-10

**Authors:** Vaishnavi Parimalanathan, Mark Joy, Pieter Jan Van Dam, Xuejuan Fan, Simon de Lusignan

**Affiliations:** 1 Tasmanian School of Medicine, College of Health and Medicine, University of Tasmania, Hobart, TAS 7000, Australia; vp16@utas.edu.au (V.P.); pieter.vandam@utas.edu.au (P.J.V.D.); 2 Nuffield Department of Primary Care Health Sciences, University of Oxford, Woodstock Road, Oxford OX2 6GG, UK; mark.joy@phc.ox.ac.uk (M.J.); xuejuan.fan@phc.ox.ac.uk (X.F.); 3 Royal College of General Practitioners Research and Surveillance Centre, 30 Euston Square, London NW1 2FB, UK

**Keywords:** influenza vaccine, general practice, vaccine hesitancy, primary care

## Abstract

Influenza, a vaccine preventable disease, is a serious global public health concern which results in a considerable burden on the healthcare system. However, vaccine hesitancy is increasingly becoming a global problem. One prevalent misconception is that influenza vaccinations can cause the flu. We carried out this study to determine whether people undertaking influenza vaccination presented less with acute respiratory tract infection (ARTI) and influenza-like-illness (ILI) following vaccination. We utilised the Oxford Royal College of General Practitioners Research and Surveillance Centre sentinel database to examine English patients who received vaccination between 2014/2015 and 2018/2019. Of the 3,841,700 influenza vaccinations identified, vaccination details and primary care respiratory consultation counts were extracted to calculate the relative incidence (RI) per exposure risk period using the self-controlled case series methodology. Results showed a significant increase in the RI of respiratory consultation rates within fourteen days of vaccination across all five years. Less than 6.2% of vaccinations led to consultations for ARTI or ILI in primary care (crude consultation rate 6196 per 100,000). These findings, particularly if confirmed in further research, may reduce the risk of cross-infection between waiting patients and increase uptake of influenza vaccine.

## 1. Introduction

Influenza is a serious global public health burden, which causes substantial morbidity and mortality ranging from mild symptoms to fatal illness worldwide each year [1,2]. It is estimated that 291,000 to 645,000 deaths are attributable to influenza annually [3]. Adults over 65 years are disproportionately affected by influenza and account for approximately 90% of all influenza-associated deaths [4]. In 2018 alone, 109.5 million influenza virus episodes and up to 34,800 overall influenza-virus-associated acute lower respiratory infection deaths were estimated to have affected children under the age of five, worldwide [5]. Annual influenza vaccination is the primary means of preventing influenza and its complications [6,7] and is recommended by the World Health Organization [8] for those most at risk of serious illness or death from influenza. Approximately 10–20% of the global population is infected with influenza viruses annually, resulting in a considerable burden on the healthcare system [9]. The burden of influenza in primary care within England [1,10,11,12] and globally [13,14] is substantial. The National Health Service (NHS) England commissions the seasonal influenza immunisation programme in England to offer protection to patients in target groups at higher risk of severe disease following infection. This includes people in a clinical at-risk group, pregnant women, children, carers, and the elderly [15]. The policy for influenza vaccination coverage is set by Public Health England prior to commencement of each influenza season and generally ranges from 50% to 75% across age and risk groups. Although the annual influenza vaccination programme is free, the uptake of vaccination has most recently fallen 3–10% [16] short of uptake ambition. In the current context of the COVID-19 pandemic, increasing vaccine uptake to reduce the impact and burden of flu illnesses on healthcare systems is vital.

Vaccine hesitancy, defined by the World Health Organization (WHO) as a “delay in acceptance or refusal of vaccines, despite availability of vaccination services” [17], is increasingly becoming a global problem. Barriers to influenza vaccination include scepticism about effectiveness, fear of side effects, and misinformation. This is particularly evident amongst healthcare personnel [18,19,20].

One prevalent misconception is that influenza vaccinations can cause the flu [21,22,23]. In England, general practitioners (GPs) provide most healthcare. For these healthcare professionals, workload is increasing [24]. Patients with flu-like symptoms seek treatment from their GP in the first instance, occurring more frequently in midwinter, at a time when the impact of other respiratory tract infections is surging [1]. There is some evidence to suggest that where people have received an influenza vaccination, the rate of GP visits is lower [1], reducing the burden on Primary Health. This is important since the impact of increasing workload on GPs can lead to health issues such as burnout [25]. It is known that the rates of burnout in GPs in the United Kingdom are higher than those in other European countries, exacerbating a healthcare system under stress [26]. To our knowledge, however, there is only a small number of studies examining the correlation between the number of people vaccinated and the rate of GP consultations. Therefore, this study has been carried out to determine whether people undertaking influenza vaccination presented less with acute respiratory tract infection (ARTI) and influenza-like-illness (ILI) following vaccination.

## 2. Materials and Methods

### 2.1. Data Source and Study Design

We conducted a retrospective cohort study using the self-controlled case series (SCCS) methodology. We utilised data from the Oxford Royal College of General Practitioners Research and Surveillance Centre (RCGP RSC), a longitudinal database of pseudonymised computerised medical records of people registered at primary care practices within this network. Clinical encounters, findings, symptoms, and diagnoses encoded using Systematized Nomenclature for Medicine—Clinical Terms (SNOMED-CT) are entered into a patient’s electronic medical record [27]. Pseudonymised patient data containing coded details of demographics, immunisation history, consultation type, and lifestyle factors are also accessible.

### 2.2. Study Period and Study Population

All patients who received the influenza vaccination during influenza season between 1 September 2014 and 31 August 2019 were identified from the Oxford RCGP RSC database. We included in this analysis cases of influenza vaccination that occurred between 1st September and 30th April of 2014–2019, which correspond to the 2014/2015, 2015/2016, 2016/2017, 2017/2018, and 2018/2019 influenza seasons. For these cases, details of valency and route of vaccination, vaccine administration date, age, and sex were extracted. Prescription data and data entered as a clinical event were used to determine valency and route of administration.

In addition, we identified primary care consultations where the presenting complaint was either ARTI or ILI using our validated case definitions and associated SNOMED CT concept lists for each. These case definitions have been utilised in several previous studies monitoring incidence of ILI/ARTI within the sentinel network [28,29,30] and underpin weekly surveillance reporting by RCGP RSC and Public Health England [31]. Dates, types, and counts of these primary care consultations were extracted into the dataset for analysis.

General practices which did not meet quality measures, patients who had chosen to “opt out” of data sharing, people not registered as permanent patients with the same practice for the entire study period or those who had died within the study period were not included. Patients who had primary care consultation date identical to vaccination date, who recorded more than one dose of influenza vaccine within the same influenza season, or who did not record vaccine valency and route were excluded.

### 2.3. Statistical Analyses

SCCS methodology was used to investigate the association between influenza vaccination and primary care consultations for respiratory infections. This case-only design yields within-person comparison, allowing each person to act as their own control [32]. The observation period of each subject is divided into risk periods, a defined time period after an exposure, and control periods, defined as time periods outside of the risk period [33,34].

Influenza vaccinations are cautioned in patients who are acutely unwell and should be postponed until the patient has fully recovered and returned to normal [35]. As it is extremely unlikely for ILI or ARTI consultation to take place immediately prior to vaccine administration, a pre-exposure risk period of 2 weeks was used. Risk period duration was determined based on existing literature on the protective effect of the influenza vaccination. A decrease in vaccine protection with increasing time since vaccination across influenza types has been observed [36]. Maximum protection has been shown 2–4 weeks after vaccination [37] followed by a significant decline around the 6-month period [36,38,39]. As such, three post-vaccination risk periods were defined [40,41].

Vaccination exposure periods, the dynamic period of interest relative to vaccination day (day 0), were defined a priori: pre-vaccination period as −1 to −14 days, post-vaccination risk period 1 as 0–14 days, risk period 2 as 15–90 days, and risk period 3 as 91–180 days (Supplementary Figure S1). Baseline period was determined by the UK influenza season, thus from 1 September to 30 April. The time period between influenza seasons, where people are not considered to be at risk of ILI or ARTI due to seasonal influenza, was defined as the washout period.

To assess seasonal effect, a 30-day rolling stratum was incorporated into the model from the 1st of October across the entire influenza season at 0–30, 31–60, 61–90, 91–120, 121–150, and 151–180 days.

We calculated the relative incidence (RI) of ARTI and ILI primary care consultations following vaccination for the different exposure periods and for the 30-day rolling stratum.

Unadjusted odds ratio (OR) was also calculated to assess the risk of respiratory consultation between live attenuated influenza vaccine (LAIV) and inactivated influenza vaccine (IIV) recipients.

All statistical analyses were performed using R Version: 3.5.3 (3 November 2019) (R Core Team (2017). F: A language and environment for statistical computing. R Foundation for Statistical Computing, Vienna, Austria. URL https://www.R-project.org/.) and SCCS package version 1.1.

## 3. Results

### 3.1. Population Characteristics

A total of 1,410,764 individuals who received influenza vaccination during the influenza seasons of 2014 to 2019 were identified. Between them, they received 3,841,700 seasonal influenza vaccinations. There was a higher prevalence of females across all five years of the study period, accounting for 54.4% in the pooled data (*n* = 2,088,730). Baseline demographic characteristics are shown in Table 1.

The age–sex profile (Supplementary Figure S2) showed a peak at 2 years with proportionally higher vaccinations up to 10 years. An increase in female vaccination between 18 and 40 years and a distinct increase at 65 years and over were also observed. This reflects the national flu immunisation programme, which provides free vaccination for eligible groups including children, pregnant women, and people over the age of 65, respectively.

### 3.2. Vaccine Type

Main types of vaccines identified across 2014/2015–2017/2018 influenza seasons were trivalent (TRI) IIV, quadrivalent (QUAD) live attenuated influenza vaccine, and QUAD IIV (Table 2). In 2018/2019, TRI IIV count dropped (*n* = 1655) as the adjuvanted trivalent inactivated influenza vaccine (aTIV) accounted for 62% (*n* = 497,758). This reflects the introduction of this new vaccine, by the Joint Committee on Vaccination and Immunisation, to the UK national flu immunisation programme as the preferred vaccine for those aged 65 years and over.

### 3.3. Primary Care Consultations

A total of 238,014 ARTI and ILI events were identified across all years of this study (Supplementary Table S1). In total, 93.7% (*n* = 222,982) were sought for ARTI, whilst 6.3% (*n* = 15,032) for ILI (Supplementary Table S2). Consultations were further subdivided by consultation type (Supplementary Table S1). Face-to-face consultations dominated (89.7%), followed by telephone consultation (8.6%), visits (1.5%). and e-consultations (0.1%).

Pooled data across all five years resulted in a crude consultation rate of 6196 per 100,000 vaccinations, with the highest crude consultation rate being recorded in the 2015/2016 season.

A fair degree of variation was observed in consultation rates by vaccine type, with LAIV accounting for the two highest proportions for primary care consultations (Table 3). Overall, over 93.8% of vaccinations did not result in primary care attendance for ARTI or ILI within the study period. After LAIV, 18.5% of individuals consulted for respiratory consultations, whilst following IIV, only 4.8% presented. The odds of LAIV recipients presenting for respiratory consultations in primary care were higher compared to the odds for those with IIV (unadjusted OR = 4.53; 95% CI = 4.49, 4.57) (Figure S3).

A total of 55.5% of vaccinated individuals were aged 65 and over (Table 1). Despite this, they accounted for the lowest presentation rate for respiratory consultations following vaccination (Supplementary Table S3). Children aged less than 5 years accounted for 5.9% of vaccinations, yet their overall respiratory consultations ranked the highest, resulting in a crude consultation rate over seven times higher than the elderly.

### 3.4. Timing of Consultations

**Figure 1:**
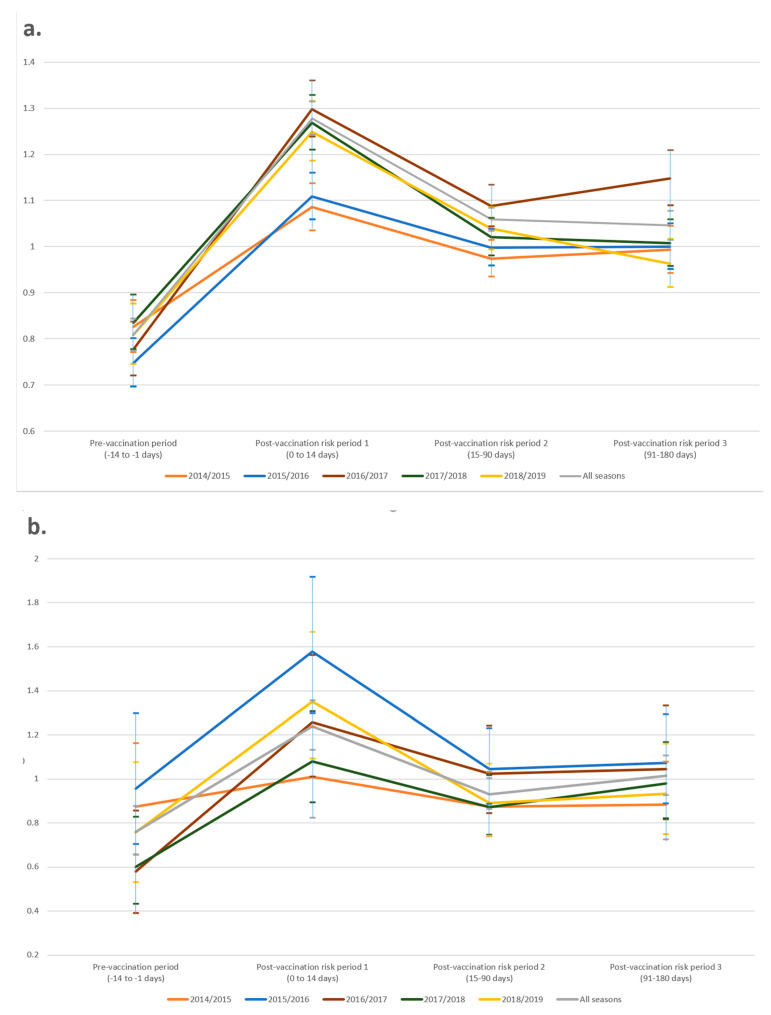
Relative incidence and 95% confidence intervals (per exposure risk period) by year for (**a**) ARTI and (**b**) ILI consultations.

Modelling showed a significant reduction in the fourteen days prior to vaccination for ARTI (combined RI = 0.81, 95% CI 0.77–0.84, *p* < 0.001) and ILI (combined RI = 0.76, 95% CI 0.66–0.88, *p* < 0.001), consistent with the healthy-vaccinee effect, across all five years. After vaccination, a significant increase in the RI of ARTI consultation rates (combined RI = 1.28, 95% CI 1.24–1.31, *p* < 0.001) within fourteen days was observed across all five years (Table 4). A similar pattern was observed for ILI consultations. Following this, RI dropped close to baseline in post-vaccination risk period 2 and was sustained for the remainder of the influenza season in post-vaccination risk period 3 (Figure 1).

**Figure 2:**
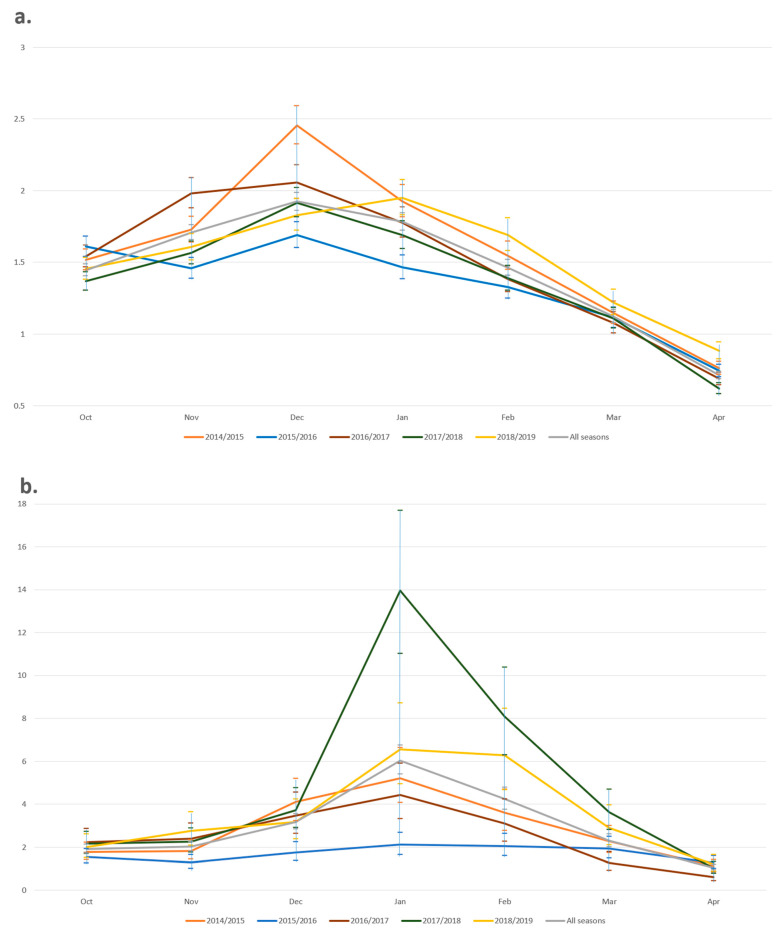
Seasonal variation in RI and 95% CI by year for (**a**) ARTI and (**b**) ILI primary care consultations.

A seasonal trend was observed in both groups of consultations (Figure 2). The RI of ARTI consultations gradually increased and peaked in December, with the exception of the 2018/2019 season, in which RI peaked in January. The RI of ILI consultations consistently peaked in January across all five years. This was most pronounced in 2017/2018, where ILI consultations were nearly thirteen times higher compared to baseline (RI = 13.96, 95% CI 11.01–17.7, *p* < 0.001).

## 4. Discussion

A high proportion of vaccine recipients did not attend primary care with a presenting complaint of ARTI or ILI in the 6 months following vaccination. Less than 6.2% of influenza vaccinations led to ARTI or ILI consultations in primary care across the rest of the influenza season (crude consultation rate of 6196 per 100,000 vaccinations).

We also observed a significant reduction in attendance at primary care in the fourteen days preceding vaccination for ARTI and ILI, consistent with the healthy-vaccinee effect, across all five years. By way of contrast, in the two-week period post-vaccination, the medical consultation rate increased, subsequently declining, then remaining close to baseline from 15 days to 6 months.

The increased consultancy rates within the fortnight after vaccination could be explained through the concept of the worried well, coupled with the misconception that a flu vaccination can cause illness. The worried well are a group of patients not being physically ill but experiencing anxiety about their health, leading to them seeking medical attention [42]. Anxiety could be one the largest issues in public health [43]. Strategies to reduce the anxiety related to flu vaccination will need to become more prominent.

Higher respiratory consultations 14 days post-vaccination could be attributable to decreased immunity and increased susceptibility to other respiratory pathogens. One study [44] demonstrated an increased hazard of acute respiratory infection caused by non-influenza respiratory pathogens in the 14 days post-vaccination compared to children who had not yet received influenza vaccination. Other studies have also identified a statistically significant increased risk of non-influenza respiratory pathogens among influenza vaccine recipients in children [45] and a higher occurrence of non-influenza ILI in vaccinated children over their unvaccinated peers [46]. Our findings show that despite accounting for less than 6% of vaccinated individuals, children aged under 5 ranked the highest for overall respiratory consultations, resulting in a crude consultation rate over seven times higher than the elderly, who accounted for 55.5% of total vaccinations. Although it has been hypothesised that vaccine-induced immunity to influenza may be accompanied by reduced immunity to non-influenza respiratory pathogens or by temporary non-specific immunity after influenza infection [45], further studies are needed to understand the intricate mechanisms of the immunological effect of influenza vaccine across all age groups and validate these plausible explanations.

A key strength of this study is its large, nationally representative dataset from a sentinel network of primary care practices within England and its offering of rapidly accessible data [47]. The use of computerised medical records containing pseudonymised details of consultations and vaccination history minimised recall bias. By employing the self-controlled case series methodology, we eliminated all time invariant confounding as individuals exposed to the vaccination acted as their own control. This allowed for underlying risks and factors such as indices of multiple deprivation, ethnicity, and sex to remain constant during the baseline and risk periods [48], making this a robust design.

There are limitations to this study. Accurate recording of vaccination date, vaccination valency/route, consultation type, and coding of clinical information are completely dependent on the health practitioner. To account for human error, patients who had vaccinations coded multiple times with the same or different clinical codes within one influenza season were excluded. However, it is possible that some patients may have received two doses of the vaccine and would have subsequently been missed in the dataset for analysis. Additionally, imprecise recording of ARTI or ILI would lead to misclassification bias. Further limitations include the number of records missing details on vaccine valency and route of administration. This resulted in the exclusion of 21.2% of records. Nonetheless, the final sample size (*n* = 3,841,700) was large enough to produce greater statistical reliability and draw strong conclusions that can be generalised to the wider population. The severity of the presenting complaint was not included in the analysis. For instance, a patient reporting mild symptoms including minor URTI would have an ARTI consultation recorded in the dataset. Important consideration must be given when interpreting this finding and drawing conclusions.

There are no comparable studies assessing primary care attendance for ARTI and ILI six months following influenza vaccination. Some studies [12,44,49] compare respiratory infections between vaccinated and unvaccinated populations. Other studies have investigated adverse outcomes following vaccinations [50,51,52]. However, to the authors’ knowledge, this is the first study to report on ARTI and ILI primary care consultations per exposure risk period. The significant elevation found in the two weeks following influenza vaccination supports the recommendation that unwell patients should not be vaccinated until fully recovered.

Seasonal trends and peaks observed in both groups of consultations are generally consistent with previous reports published by UK national bodies [16,53,54,55,56]. In 2017/2018, moderate to high levels of influenza were reported by Public Health England [56], and overall all-age adjusted vaccine effectiveness in the UK was approximated at 15% against all influenza [57]. This is reflected in our data, which show a significant increase in RI of ILI consultations at 13.96 compared to baseline.

We observed a greater rate of respiratory consultations following LAIV. However, given we did not adjust for the propensity to consult in children [58] in the age groups receiving LAIV, this may not be a true difference.

The study findings show that an exceedingly high proportion of influenza vaccine recipients do not attend primary care for respiratory consultation following vaccine administration. Primary care workers have a key role in helping patients to understand the benefits of vaccination; hence, they may use these findings to dispel misconceptions and increase vaccination uptake across all target groups. However, not knowing the exact impact of flu vaccination on children, further evaluation studies of the advantages and disadvantages of vaccinating for this cohort should be undertaken.

A common misconception leading to vaccine hesitancy is that influenza vaccinations can cause the flu. This has been highlighted as a prominent issue over the years. Taking into account that less than 6.2% of the vaccinations led to consultations for ARTI or ILI in primary care, more work is required to alleviate this misconception. Incorporating this finding into international campaigns to promote the salience of this beneficial outcome and to strengthen the positive association with vaccination may help to reduce misconceptions [59] and directly support public health policies.

In addition to this, recognising the significance of the fortnight following vaccination has the potential to transform patient education and care for vaccine recipients around the world. Counselling and reassuring patients of potential symptoms and encouraging self-management for mild, transient symptoms within this exposure period could significantly impact primary care workload, particularly when the greatest burden on general practitioner services in England within respiratory diseases is associated with influenza [24]. This is pertinent this winter whilst influenza and COVID-19 are co-circulating.

## 5. Conclusions

The relative incidence of ARTI and ILI primary care consultations was elevated during the two-week period following influenza vaccination. Despite this, a large proportion of influenza vaccine recipients did not return to primary care with a presenting complaint of ARTI or ILI within 6 months of vaccination. These findings, if confirmed in other studies, might be a positive factor to help to reduce vaccine hesitancy and considered when educating and treating vaccine recipients, as well as informing vaccination policy. Reduction in consultations for ARTI and ILI may reduce the chance of cross-infection between patients. Further research is required to confirm our findings that influenza vaccination may reduce the consultation burden from acute reception on general practice and primary care.

## Figures and Tables

**Table 1:** Demographic characteristics of seasonal influenza vaccine recipients in the Oxford Royal College of General Practitioners Research and Surveillance Centre (RCGP RSC) network between 2014/2015 and 2018/2019.

	2014/2015		2015/2016		2016/2017		2017/2018		2018/2019		All Years	
**Participants**	702,402		750,819		783,157		802,001		803,321		3,841,700	
**Sex (%)**												
Female	385,186	(54.8)	409,582	(54.6)	424,536	(54.2)	435,135	(54.3)	434,291	(54.1)	2,088,730	(54.4)
Male	317,216	(45.2)	341,237	(45.4)	358,621	(45.8)	366,866	(45.7)	369,030	(45.9)	1,752,970	(45.6)
**Age Band (%)**												
<1	566	(0.1)	348	(0)	0	(0)	0	(0)	0	(0)	914	(0)
1–4	51,980	(7.4)	63,324	(8.4)	61,750	(7.9)	35,120	(4.4)	15,480	(1.9)	227,654	(5.9)
5–9	10,953	(1.6)	22,096	(2.9)	29,722	(3.8)	35,178	(4.4)	44,403	(5.5)	142,352	(3.7)
10–14	10,965	(1.6)	9084	(1.2)	9569	(1.2)	10,518	(1.3)	10,117	(1.3)	50,253	(1.3)
15–19	5782	(0.8)	6117	(0.8)	6633	(0.8)	7708	(1)	7529	(0.9)	33,769	(0.9)
20–24	7628	(1.1)	7478	(1)	7170	(0.9)	7356	(0.9)	6595	(0.8)	36,227	(0.9)
25–29	11,289	(1.6)	11,060	(1.5)	10,682	(1.4)	10,669	(1.3)	8,971	(1.1)	52,671	(1.4)
30–34	15,079	(2.1)	14,592	(1.9)	14,123	(1.8)	14,277	(1.8)	12,162	(1.5)	70,233	(1.8)
35–39	15,926	(2.3)	15,930	(2.1)	15,937	(2)	16,534	(2.1)	14,930	(1.9)	79,257	(2.1)
40–44	21,759	(3.1)	21,568	(2.9)	21,038	(2.7)	21,446	(2.7)	18,901	(2.4)	104,712	(2.7)
45–49	28,825	(4.1)	29,130	(3.9)	29,380	(3.8)	31,333	(3.9)	29,072	(3.6)	147,740	(3.9)
50–54	35,914	(5.1)	37,061	(4.9)	38,065	(4.9)	40,962	(5.1)	38,991	(4.9)	190,993	(5)
55–59	42,264	(6)	43,520	(5.8)	45,624	(5.8)	48,899	(6.1)	47,963	(6)	228,270	(5.9)
60–64	69,334	(9.9)	67,642	(9)	67,858	(8.7)	71,451	(8.9)	69,384	(8.6)	345,669	(9)
65–69	130,530	(18.6)	131,239	(17.5)	132,257	(16.9)	132,361	(16.5)	124,932	(15.6)	651,319	(17)
70–74	100,488	(14.3)	107,254	(14.3)	112,618	(14.4)	121,626	(15.2)	134,622	(16.8)	576,608	(15)
75–79	77,286	(11)	84,202	(11.2)	88,337	(11.3)	90,357	(11.3)	95,964	(11.9)	436,146	(11.4)
80–84	44,375	(6.3)	51,215	(6.8)	57,330	(7.3)	62,973	(7.9)	69,811	(8.7)	285,704	(7.4)
85–89	17,170	(2.4)	21,677	(2.9)	26,373	(3.4)	31,553	(3.9)	37,780	(4.7)	134,553	(3.5)
90–94	3996	(0.6)	5772	(0.8)	7659	(1)	9991	(1.2)	13,069	(1.6)	40,487	(1.1)
95+	293	(0)	510	(0.1)	1032	(0.1)	1689	(0.2)	2645	(0.3)	6169	(0.2)

**Table 2:** Rates of influenza vaccination by vaccine type between 2014/2015 and 2018/2019.

	2014/2015		2015/2016		2016/2017		2017/2018		2018/2019		All Years	
**Valency, Route (%)**												
TRI, LAIV	720	(0.1)	43	(0)	0	(0)	0	(0)	0	(0)	763	(0)
TRI, IIV	627,720	(89.4)	646,975	(86.2)	65,2191	(83)	466,050	(58.1)	1655	(0.2)	2,394,591	(62.3)
QUAD, LAIV	66,605	(9.5)	88,809	(11.8)	96,617	(12)	77,192	(9.6)	68,783	(8.6)	398,006	(10.4)
QUAD, IIV	7357	(1)	14,991	(2)	34,349	(4)	258,759	(32.3)	235,125	(29.3)	550,581	(14.3)
aTIV, IIV	0	(0)	1	(0)	0	(0)	0	(0)	497,758	(62)	497,759	(13)
**Total**	702,402		750,819		783,157		802,001		803,321		3,841,700	

**Table 3:** Primary care consultations by vaccine type across all influenza seasons (2014/2015 to 2018/2019).

	ARTI Consultations		ILI Consultations		No Consultations		Total
Vaccine Valency, Route (%)							
TRI, LAIV	79	(10.3)	2	(2.6)	682	(89.4)	763
TRI, IIV	106,850	(4.5)	9946	(0.4)	2,277,795	(95.1)	2,394,591
QUAD, LAIV	73,042	(18.4)	649	(0.2)	324,315	(81.5)	398,006
QUAD, IIV	28,912	(5.3)	2947	(0.5)	518,722	(94.2)	550,581
aTIV, IIV	14,099	(2.8)	1488	(0.3)	482,172	(96.9)	497,759
All vaccinations	222,982	(5.8)	15,032	(0.4)	3,603,686	(93.8)	3,841,700

**Table 4:** Relative incidence (RI) of acute respiratory tract infections (ARTI) and influenza-like illness (ILI) consultations for exposure risk periods.

Year	Exposure Risk Period	ARTI			ILI		
		RI	CI (95%)	*p*-Value	RI	CI (95%)	*p*-Value
	**Pre-vaccination** **−14 to −1 days**						
2014/2015		0.83	0.77–0.88	<0.001	0.87	0.66–1.16	0.36
2015/2016		0.75	0.70–0.80	<0.001	0.96	0.70–1.30	0.77
2016/2017		0.78	0.72–0.84	<0.001	0.58	0.39–0.86	0.0062
2017/2018		0.83	0.78–0.90	<0.001	0.60	0.43–0.83	0.0019
2018/2019		0.81	0.75–0.88	<0.001	0.76	0.53–1.08	0.12
All years (2014–2019)		0.81	0.77–0.84	<0.001	0.76	0.66–0.88	<0.001
	**Post-vaccination** **0–14 days**						
2014/2015		1.09	1.04–1.14	<0.001	1.01	0.82–1.24	0.93
2015/2016		1.11	1.06–1.16	<0.001	1.58	1.30–1.92	<0.001
2016/2017		1.30	1.24–1.36	<0.001	1.26	1.01–1.56	0.039
2017/2018		1.27	1.21–1.33	<0.001	1.08	0.89–1.31	0.43
2018/2019		1.25	1.10–1.32	<0.001	1.35	1.09–1.67	0.0054
All years (2014–2019)		1.28	1.24–1.31	<0.001	1.24	1.13–1.36	<0.001
	**Post-vaccination** **15–90 days**						
2014/2015		0.97	0.93–1.01	0.20	0.87	0.74–1.03	0.11
2015/2016		1.00	0.96–1.04	0.92	1.04	0.89–1.23	0.60
2016/2017		1.09	1.04–1.13	<0.001	1.02	0.84–1.24	0.81
2017/2018		1.02	0.98–1.06	0.31	0.87	0.75–1.02	0.08
2018/2019		1.04	0.99–1.09	0.089	0.89	0.74–1.07	0.21
All years (2014–2019)		1.06	1.03–1.08	<0.001	0.93	0.86–1.00	0.06
	**Post-vaccination** **91–180 days**						
2014/2015		0.99	0.94–1.05	0.79	0.88	0.72–1.08	0.22
2015/2016		1.00	0.95–1.05	0.99	1.07	0.89–1.29	0.47
2016/2017		1.15	1.09–1.21	<0.001	1.04	0.82–1.33	0.73
2017/2018		1.01	0.96–1.06	0.77	0.98	0.82–1.17	0.81
2018/2019		0.96	0.91–1.02	0.18	0.93	0.75–1.16	0.53
All years (2014–2019)		1.05	1.02–1.08	0.0034	1.01	0.93–1.11	0.77

This table shows the RI of ILI and ARTI consultations occurring in time periods −14 to −1, 0 to 14, 15 to 90, and 91 to 180 days after vaccination (Day 0). A relative incidence of 1 indicates no change from baseline.

## Data Availability

Oxford-RCGP RSC data are available for independent scientific research. Applications for data access are made via www.rcgp.org.uk/rsc.

## Supplementary Materials

The following are available online at https://www.mdpi.com/1660-4601/18/2/523/s1, Figure S1: Influenza season (1 September to 30 April) divided into baseline, pre-vaccination, and post-vaccination risk periods. Baseline extended from 1 September to 15 days pre-vaccination and from 180 days postvaccination to 30 April. Washout period (non-influenza season) extended from 1st May to 31st of August, Figure S2: Age-sex profile for seasonal influenza vaccine recipients in the Oxford RCGP RSC network between 2014/2015 to 2018/2019, Figure S3: Vaccine type by respiratory consultation (2-by-2 table). Table S1: Total number of reported ARTI and ILI events within influenza season between 2014/15 to 2018/19. Table S2: Total count of consultations by consultation type across all influenza seasons for 2014 to 2019. Table S3: Vaccination and respiratory consultations, stratified by age.
