# Supplementary material for: Association between Influenza Vaccine Administration and Primary Care Consultations for Respiratory Infections: Sentinel Network Study of Five Seasons (2014/2015–2018/2019) in the UK
Source: Int J Environ Res Public Health. 2021 Jan 10;18(2):523. doi: 10.3390/ijerph18020523 (PMC7827078; doi:10.3390/ijerph18020523)
Supplement: Supplementary file 1 [file ijerph-18-00523-s001.pdf]

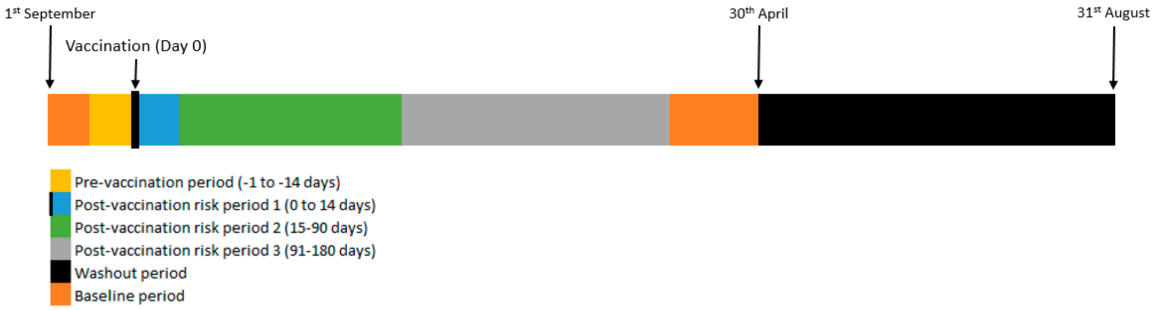

Figure S1. Influenza season (1 September to 30 April) divided into baseline, pre-vaccination, and post-vaccination risk periods. Baseline extended from 1 September to 15 days pre-vaccination and from 180 days postvaccination to 30 April. Washout period (non-influenza season) extended from 1st May to 31st of August.

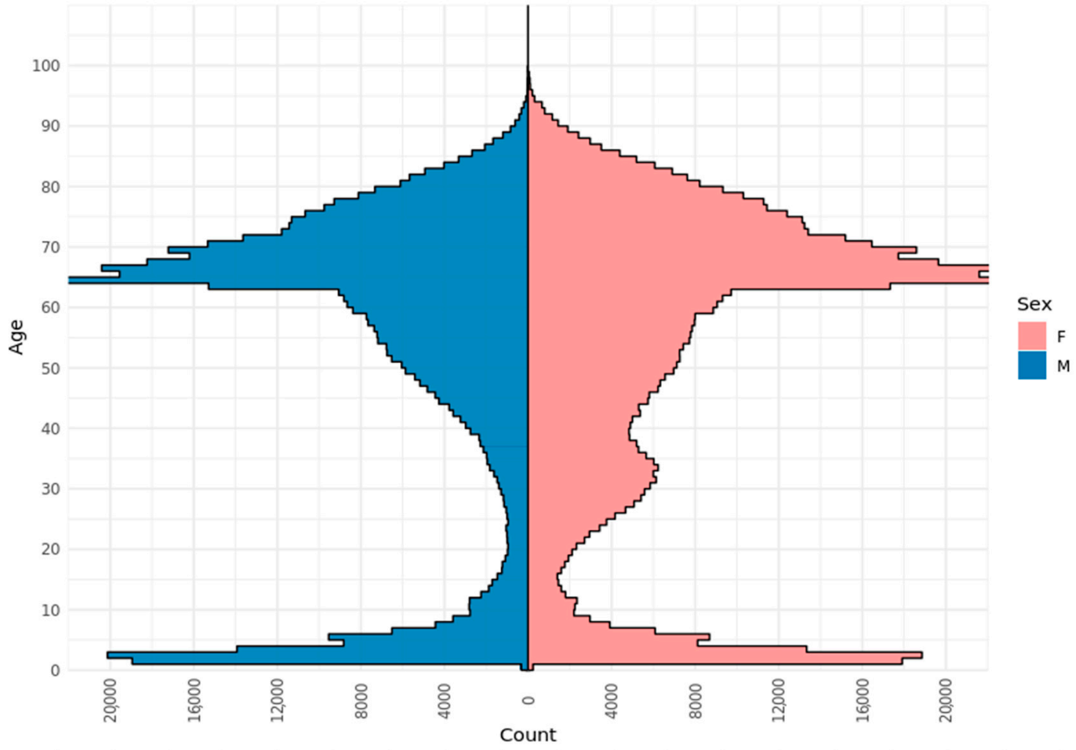

Figure S2. Age-sex profile for seasonal influenza vaccine recipients in the Oxford RCGP RSC network between 2014/2015 to 2018/2019.

	Respiratory consultation	No respiratory consultation
**LAIV**	73,772	324,997
**IIV**	164,242	3,278,689

Figure S3. Vaccine type by respiratory consultation (2-by-2 table).

Table S1. Total count of consultations by consultation type across all influenza seasons for 2014 to 2019.

	2014/2015		2015/2016		2016/2017		2017/2018		2018/2019		All Years	
**Consultation Type (%)**												
E-Consultation	1	(0)	2	(0)	6	(0)	67	(0.1)	177	(0.5)	253	(0.1)
Face to Face	34,907	(89.5)	50,593	(90.2)	49,251	(90.3)	44,164	(89.3)	34,852	(89.5)	213,767	(89.7)
Telephone Consultation	3523	(9)	4733	(8.4)	4555	(8.4)	4331	(8.8)	3057	(7.8)	20,199	(8.6)
Visit	556	(1.4)	746	(1.3)	725	(1.3)	905	(1.8)	863	(2.2)	3795	(1.5)
Total	38,987		56,074		54,537		49,467		38,949		238,014	

Table S2. Total number of reported ARTI and ILI events within influenza season between 2014/2015 to 2018/2019.

	2014/2015		2015/2016		2016/2017		2017/2018		2018/2019		All Years	
**Disease name (%)**												
Acute Respiratory Tract Infections	36,547	(93.7)	53,228	(94.9)	51,812	(95)	45,446	(91.9)	35,949	(92.3)	222,982	(93.7)
Influenza-like illness	2440	(6.3)	2846	(5.1)	2725	(5)	4021	(8.1)	3000	(7.7)	15,032	(6.3)
Total	38,987		56,074		54,537		49,467		38,949		238,014	
**Crude consultation rates/100,000 vaccinations**	5551		7468		6964		6168		4848		6196	

Table S3. Vaccination and respiratory consultations, stratified by age.

Age Band	Vaccinations	Respiratory Consultations	Crude Consultation Rates/100,000 Vaccinations
<5	228,568	55,537	24,297
5–19	226,374	27,589	12,187
20–49	490,840	44,383	9042
50–64	764,932	39,383	5149
65+	2,130,986	71,122	3338
Total	3,841,700	238,014	6196
